# Intranasal neomycin evokes broad-spectrum antiviral immunity in the upper respiratory tract

**DOI:** 10.1073/pnas.2319566121

**Published:** 2024-04-22

**Authors:** Tianyang Mao, Jooyoung Kim, Mario A. Peña-Hernández, Gabrielee Valle, Miyu Moriyama, Sophia Luyten, Isabel M. Ott, Maria Luisa Gomez-Calvo, Jeff R Gehlhausen, Emily Baker, Benjamin Israelow, Martin Slade, Lokesh Sharma, Wei Liu, Changwan Ryu, Asawari Korde, Chris J. Lee, Valter Silva Monteiro, Carolina Lucas, Huiping Dong, Yi Yang, Yale SARS-CoV-2 Genomic Surveillance Initiative, Smita Gopinath, Craig B. Wilen, Noah Palm, Charles S. Dela Cruz, Akiko Iwasaki

**Affiliations:** ^a^Department of Immunobiology, Yale University School of Medicine, New Haven, CT 06510; ^b^Department of Internal Medicine, Section of Pulmonary, Critical Care and Sleep Medicine, Yale University School of Medicine, New Haven, CT 06510; ^c^Division of Pulmonary, Allergy, Critical Care, and Sleep Medicine, Department of Medicine, University of Pittsburgh, Pittsburgh PA 15213; ^d^Department of Microbial Pathogenesis, Yale University School of Medicine, New Haven CT 06510; ^e^Department of Dermatology, Yale University School of Medicine, New Haven, CT 06510; ^f^Department of Internal Medicine, Section of Infectious Diseases, Yale University School of Medicine, New Haven, CT 06510; ^g^Department of Internal Medicine, Section of Occupational Medicine, Yale University School of Medicine, New Haven, CT 06510; ^h^Department of Immunology and Infectious Diseases, Harvard T.H. Chan School of Public Health, Harvard University, Boston, MA 02115; ^i^Department of Laboratory Medicine, Yale University School of Medicine, New Haven, CT 06510; ^j^Veterans Affairs Pittsburgh Healthcare System, Pittsburgh, PA 15240; ^k^Center for Infection and Immunity, Yale University School of Medicine, New Haven, CT 06510; ^l^HHMI, Chevy Chase, MD 20815

**Keywords:** nasal, interferon, antiviral, mucosal immunity, transmission

## Abstract

Respiratory virus infections in humans cause a broad-spectrum of diseases that result in substantial morbidity and mortality annually worldwide. To reduce the global burden of respiratory viral diseases, preventative and therapeutic interventions that are accessible and effective are urgently needed, especially in countries that are disproportionately affected. Repurposing generic medicine has the potential to bring new treatments for infectious diseases to patients efficiently and equitably. In this study, we found that intranasal delivery of neomycin, a generic aminoglycoside antibiotic, induces the expression of interferon-stimulated genes (ISGs) in the nasal mucosa that is independent of the commensal microbiota. Prophylactic or therapeutic administration of neomycin provided significant protection against upper respiratory infection and lethal disease in a mouse model of COVID-19. Furthermore, neomycin treatment protected Mx1 congenic mice from upper and lower respiratory infections with a highly virulent strain of influenza A virus. In Syrian hamsters, neomycin treatment potently mitigated contact transmission of severe acute respiratory syndrome coronavirus 2 (SARS-CoV-2). In healthy humans, intranasal application of neomycin-containing Neosporin ointment was well tolerated and effective at inducing ISG expression in the nose in a subset of participants. These findings suggest that neomycin has the potential to be harnessed as a host-directed antiviral strategy for the prevention and treatment of respiratory viral infections.

## Significance

Respiratory virus infections in humans are a significant global health concern, causing a wide range of diseases with substantial morbidity and mortality worldwide. This underscores the urgent need for effective interventions to reduce the burden of respiratory viral diseases, especially in countries disproportionately affected. In this work, we repurpose a widely available generic medication, neomycin, as a host-directed antiviral strategy to combat upper respiratory viral infection and transmission, offering hope for addressing a significant global health challenge.

Human respiratory viruses include diverse families of viral pathogens that establish infection in the respiratory tract, elicit respiratory symptoms, and transmit primarily via aerosolized and droplet respiratory secretions from infected individuals. The detrimental impact of respiratory viruses on humanity was exemplified by the global COVID-19 pandemic caused by a newly emerged coronavirus SARS-CoV-2. The pandemic has thus far infected 774.5 million people with global mortality of 6.9 million as of February 2024. In addition to pathogenic human coronaviruses, respiratory infection with influenza viruses continues to cause morbidity and mortality in the human population, accounting for up to 5 million cases of severe illness and 500,000 deaths annually worldwide (1, 2). Respiratory viral infections typically begin in the upper respiratory tract where viral pathogens infect the epithelium of the nasal cavity and nasopharynx to cause mild illness. However, failure to contain the upper respiratory infection by the host can lead to the further spread of viruses to the lower respiratory tract, resulting in more severe forms of the disease such as acute respiratory distress syndrome that can become fatal. Despite impressive advances in diagnostics, treatment, vaccines, and public health policies, there remains an unmet need for adjunct approaches to limit the impact of respiratory viral pathogens. Furthermore, currently available medicines for viral infections are not equitably accessible, particularly in low-income countries where access to essential medicines has historically been limited.

Most therapeutic interventions for respiratory viral infections focus on mitigating disease progression once infections have been established. Examples include antivirals, monoclonal antibodies, convalescent plasma therapy, and immunomodulatory agents. These therapeutics are administered systemically or orally. In contrast, therapeutics administered locally to the respiratory tract via an intranasal route aim to block infection altogether, before the virus has a chance to spread to the lower respiratory tract and cause severe or chronic diseases. Successful containment of viral infection in the upper respiratory tract also has important implications for transmission mitigation. By reducing viral loads in the upper airway, topical medicines can limit the viral load being transmitted from infected donors to exposed recipients (3). Altogether, strengthening antiviral protection at the upper respiratory mucosa holds substantial promise for ameliorating the burden of viral diseases.

Antiviral therapeutics can be divided into two major categories based on their mechanisms of action. An effective antiviral may either interfere with the viral life cycle by directly antagonizing the virus (virus-directed), or by mobilizing the host immune system to restrict viral infection (host-directed) (4, 5). Growing evidence suggests that the innate immune system can be pharmacologically targeted to elicit a protective antiviral program (6). This program is characterized by the concerted transcriptional induction of hundreds of interferon-stimulated genes (ISGs). ISGs are a broad family of effector proteins induced by interferons (IFNs) that interfere with different stages of the viral life cycle, providing a robust first line of defense against invading viruses (7). In individuals infected with respiratory viruses, the inability to mount a timely and effective ISG response is associated with delayed viral clearance and susceptibility to severe disease (8–10). However, therapeutic strategies that trigger ISG-based antiviral immunity in the upper respiratory mucosa are poorly explored.

In a previous effort to better understand the host effects of commonly used antibiotics, we have reported that intravaginal application of aminoglycoside antibiotics increased host resistance to genital infections with DNA and RNA viruses (11). Aminoglycoside-mediated antiviral protection occurred in germfree (GF) mice, suggesting a microbiota-independent mechanism of resistance. Importantly, the antiviral property of aminoglycoside antibiotics hinges on their ability to trigger the expression of ISGs in the genital mucosa. Aminoglycosides also conferred disease protection against lethal influenza infection in mice when administered intranasally, likely through restricting viral infection in the lower respiratory tract (11). However, whether aminoglycosides engage an ISG-mediated antiviral response in the upper respiratory mucosa, and whether such induction occurs in other animals and humans remains undetermined. In this study, we assessed whether intranasal application of neomycin, an aminoglycoside antibiotic, evokes ISG-based antiviral protection against SARS-CoV-2 and influenza A virus infection in the upper respiratory tract in mice, and whether nasal neomycin application confers transmission blockade in hamsters. We further investigated whether intranasal topical application of Neosporin, a neomycin-containing antibiotic ointment, induces ISG expression in the nasal mucosa in humans.

## Intranasal Neomycin Treatment Induces an ISG Response in the Nasal Mucosa that Is Independent of Commensal Microbiota

**Fig. 1.**
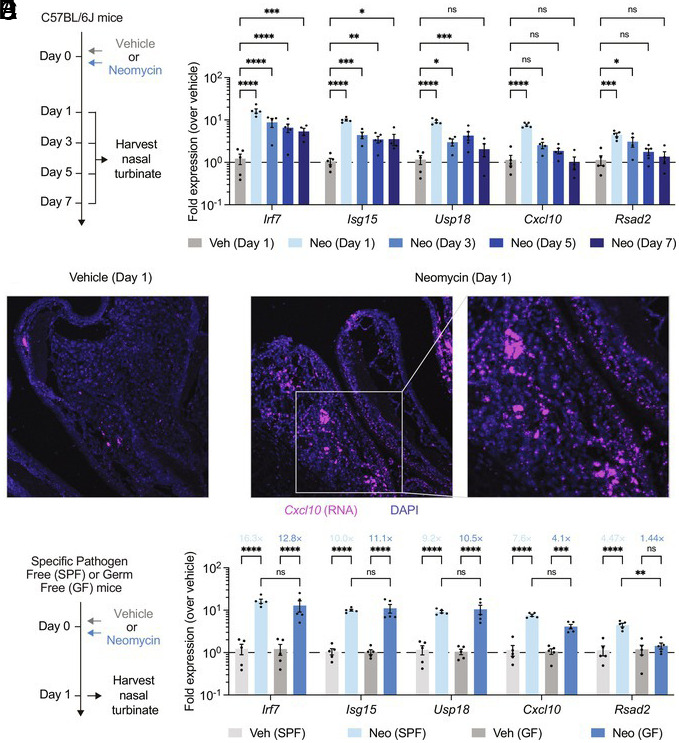
Intranasal application of neomycin induces an upper respiratory ISG response independent of commensal microbiota. (*A*) Experimental schema. Specific-pathogen-free (SPF) C57BL/6J mice were intranasally treated once with 2 mg neomycin, or vehicle delivered in 10 μL volume per nostril. On days 1, 3, 5, and 7 posttreatment, nasal turbinates were collected for gene expression analysis by RT-qPCR and for RNA FISH analysis (for day 1 tissues only). (*B*) Expression of ISGs *Irf7*, *Isg15*, *Usp18*, *Cxcl10*, and *Rsad2* in nasal turbinate tissues (Day 1, n = 5; Day 3, n = 4; Day 5, n = 5; Day 7, n = 4). Gene expression was normalized against housekeeping genes *Hprt*, and then compared against biological controls (vehicle-treated mice). (*C*) RNA FISH analysis of *Cxcl10* expression in paraffin-embedded nasal turbinate tissues. (*D*) Experimental schema. SPF or GF mice were intranasally treated once with 2 mg neomycin or vehicle. On day 1 posttreatment, nasal turbinates were collected for gene expression analysis by RT-qPCR. (*E*) Expression of ISGs *Irf7*, *Isg15*, *Usp18*, *Cxcl10*, and *Rsad2* in nasal turbinate tissues (SPF + Vehicle, n = 5; SPF + Neomycin, n = 5; GF + Vehicle, n = 5; GF + Neomycin, n = 5). Gene expression of SPF and GF samples were separately compared to their vehicle controls. To reduce the overall number of experimental animals used, control data points from naïve and neomycin-treated animals housed in the SPF facility are common to *B* and *E*. For RNA FISH analyses, representative staining results were shown. Sections are representative of multiple sections from at least five mice per group. Mean ± SEM; statistical significance was calculated by means of two-way ANOVA followed by Tukey’s correction (*B* and *E*); *P ≤ 0.05, **P ≤ 0.01, ***P ≤ 0.001, ****P ≤ 0.0001. Individual data points are represented. Samples from different timepoints were collected from different animals, not the same animals longitudinally.

To investigate whether local neomycin treatment induces ISG expression in the upper airway, we treated mice with a single dose of 2 mg neomycin sulfate (neomycin in short hereafter) administered intranasally. We chose a low-volume inoculation protocol in which only 10 μL of neomycin solution per nostril was administered, restricting the distribution of neomycin inside the nasal cavity (12). On days 1, 3, 5, and 7 after neomycin treatment, mice were euthanized from which nasal turbinate tissues were collected (Fig. 1*A*). Compared to vehicle controls, neomycin-treated mice had significantly increased levels of ISG expression in the nose (Fig. 1*B*). Neomycin-induced ISG response was rapid, as it occurred as early as 1 d after a single-dose treatment. Following the initial induction, distinct longitudinal expression patterns were observed among different ISGs. *Irf7*, *Isg15,* and *Usp18* were more persistently induced, maintaining their expression for up to 5 d posttreatment, whereas *Cxcl10* and *Rsad2* were more short-lived. We independently confirmed the upregulation of *Cxcl10* using RNA fluorescence in situ hybridization (RNA FISH), which revealed its widespread induction in the epithelium and the underlying lamina propria of the nasal mucosa (Fig. 1*C*). These results are consistent with our previous findings in the vaginal and lower respiratory mucosa and suggest that neomycin rapidly mobilizes an antiviral program in the upper respiratory mucosa.

The impact of antibiotics on host immunity is often attributed to the perturbation of susceptible commensal bacterial communities. To assess whether neomycin-mediated ISG response is dependent on the host microbiota, we treated GF mice intranasally with neomycin. Nasal turbinate tissues were collected 1 d posttreatment for assessment of ISG expression (Fig. 1*D*). Notably, neomycin treatment of GF hosts resulted in a robust upper respiratory ISG response that was comparable to that observed in specific-pathogen-free (SPF) mice, with the exception of *Rsad2* (Fig. 1*E*). These data indicate that the ability of neomycin to induce ISG expression in the upper respiratory tract is largely independent of commensal bacteria.

## Neomycin-Triggered ISG Response Does Not Require Type I or III IFN Receptor Signaling

At barrier tissues, induction of ISGs typically requires signaling through IFN receptors IFNAR (for type I IFNs) or IFNLR (for type III IFNs). To examine whether neomycin-induced ISG expression requires IFNs, we measured the secretion of IFN-α (type I), IFN-β (type I), and IFN-λ (type III) in the nasal cavity (*SI Appendix*, Extended Fig. 1*A*). Interestingly, none of the IFNs were found to be significantly induced following neomycin treatment despite the use of high-sensitivity detection methods (*SI Appendix*, Fig. S1 *B*–*D*). Consistent with this, neomycin-mediated ISG induction was intact in mice lacking either IFNAR or IFNLR (*SI Appendix*, Fig. S1*E*), suggesting that these signaling pathways are dispensable. These results suggest that neomycin promotes ISG expression in an IFN-independent manner, or that IFNAR and IFNLR play redundant roles. A recent study discovered that interleukin-1 (IL-1) family cytokines, in particular IL-1α, is capable of inducing ISG expression in the barrier epithelia (13). Additionally, the type II IFN-γ is known to trigger ISGs and contribute to antiviral immune responses (7). To assess whether these cytokines are involved in neomycin-triggered antiviral immunity, we measured the levels of IL-1α and IFN-γ in nasal washes from neomycin-treated animals. Intranasal treatment with neomycin did not induce detectable changes of IL-1α in nasal washes at either 6 or 24 h posttreatment (*SI Appendix*, Fig. S1*F*), suggesting that IL-1α was not required for the observed ISG response. Interestingly, there was a transient increase of IFN-γ levels at 6 h post neomycin treatment (*SI Appendix*, Fig. S1*G*), which suggests that IFN-γ might be involved in ISG induction.

## Single-Dose Intranasal Neomycin Prophylaxis Affords Antiviral Protection against SARS-CoV-2 Infection and Disease

**Fig. 2.**
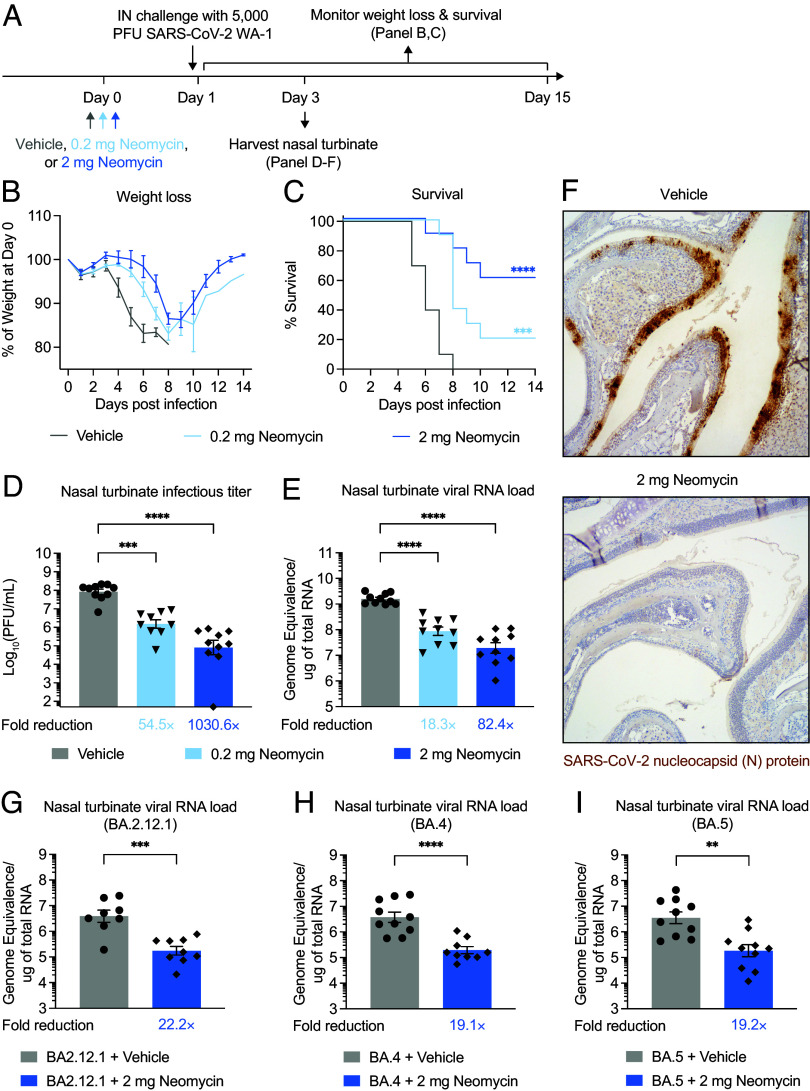
Neomycin prophylaxis affords antiviral protection against SARS-CoV-2 infection and disease. (*A*) Experimental schema. K18-hACE2 mice housed in a SPF facility were intranasally treated once with 0.2 mg neomycin, 2 mg neomycin or vehicle delivered in 10 μL volume per nostril. One day after treatment, mice were intranasally infected with 5 × 10^3^ PFU SARS-CoV-2 (2019n-CoV/USA_WA1/2020). In one group of mice, weight loss and survival were monitored daily up to 14 DPI. Death was recorded when mice were moribund, or at 80% of original body weight. In a separate cohort, nasal turbinate tissues were collected for viral titer analysis 2 DPI. (*B* and *C*) Weight loss and survival of K18-hACE2 mice prophylactically treated with vehicle, 0.2 mg neomycin, or 2 mg neomycin from 1 to 14 DPI (Vehicle, n = 10; 0.2 mg Neomycin, n = 10; 2 mg Neomycin, n = 10). (*D*) Measurement of infectious virus titer in the nasal turbinate at 2 DPI by plaque assay (Vehicle, n = 10; 0.2 mg Neomycin, n = 9; 2 mg Neomycin, n = 10). Limit of detection (LOD): 10^2^ PFU/mL. (*E*) Measurement of vRNA in the nasal turbinate at 2 DPI by RT-qPCR against SARS-CoV-2 N gene using the CDCN1 primer-probe set (Vehicle, n = 10; 0.2 mg Neomycin, n = 10; 2 mg Neomycin, n = 10). (*F*) Representative images of immunohistochemistry staining of SARS-CoV-2 N protein on nasal turbinate sections from vehicle- or neomycin-treated mice. (*G*–*I*) Measurement of vRNA in the nasal turbinate at 2 DPI from mice infected with BA.2.12.1, BA.4 or BA.5 by RT-qPCR against SARS-CoV-2 N gene using the CDCN1 primer-probe set (BA2.12.1 + Vehicle, n = 8; BA2.12.1 + Neomycin, n = 9; BA.4 + Vehicle, n = 10; BA.4 + Neomycin, n = 9; BA.5 + Vehicle, n = 10; BA.5 + Neomycin, n = 10). Fold reduction was calculated as average fold reduction in infectious (*D*) or genomic RNA (*E* and *G*–*I*) virus load between neomycin-treated groups and vehicle controls. For histological analyses, representative H&E staining results were shown. Sections are representative of multiple sections from at least five mice per group. Mean ± SEM; statistical significance was calculated by the log-rank Mantel–Cox test (*C*), one-way ANOVA followed by Tukey correction (*D* and *E*) or Student's *t* test (*G*–*I*). Individual data points are represented. Data are pooled from two independent experiments.

To determine whether neomycin-mediated ISG induction in the upper airway has any functional consequence on respiratory virus infection, we used a transgenic mouse model of SARS-CoV-2 infection. In this model, the expression of human angiotensin-converting enzyme 2 (hACE2) is under the control of a keratin 18 (K18) gene promoter, enabling epithelial infection by SARS-CoV-2 (14, 15). Intranasal exposure to the virus in K18-hACE2 mice causes robust infection of the upper and lower airway that rapidly progresses into lethal disease, characterized by weight loss, display of sickness behaviors, lethargy, and ultimately death. We first intranasally treated mice with either 2 or 0.2 mg of neomycin sulfate (Fig. 2*A*). One day after neomycin treatment, mice were infected with the ancestral strain of SARS-CoV-2 (2019n-CoV/USA_WA1/2020). Neomycin treatment considerably prevented weight loss and improved survival following viral infection in a dose-dependent manner, with the 2 mg dosing regimen mediating more apparent disease protection (Fig. 2 *B* and *C*). Nevertheless, a subset of mice receiving 0.2 mg of neomycin was protected from weight loss and lethality following viral challenge. In contrast, vehicle-treated mice uniformly lost weight and succumbed to infection by 8 days postinfection (DPI). On 2 DPI, neomycin-treated mice had lower viral replication in nasal tissues evidenced by significantly reduced levels of infectious virus (Fig. 2*D*) and viral genomic RNA (vRNA) (Fig. 2*E*). Consistent with disease protection, the 2 mg dose group led to a more pronounced reduction of viral titers than the 0.2 mg dose group. We further validated upper respiratory viral control by immunohistochemical staining of the SARS-CoV-2 nucleocapsid (N) protein. There were substantially reduced levels of N protein in nasal turbinate tissues from neomycin-treated mice compared to vehicle controls (Fig. 2*F*). Collectively, these data indicate that prophylactic intranasal application of neomycin reduces upper respiratory infection and lethal disease in a mouse model of SARS-CoV-2.

Next, we examined whether prophylactic neomycin treatment is protective against infections with SARS-CoV-2 variants. We focused on 3 recently emerged omicron variants, BA2.12.1, BA.4, and BA.5. Mice were intranasally treated with neomycin and 1 d later challenged with the omicron variants. On 2 DPI, nasal turbinate tissues were collected for viral titer assessment. Notably, neomycin-treated mice had significantly lower levels of SARS-CoV-2 vRNA in their nasal tissues, suggesting suppressed viral replication (Fig. 2 *G*–*I*). These results indicate that neomycin prophylaxis affords antiviral protection against phylogenetically diverse SARS-CoV-2 strains in mice.

## Intranasal Neomycin Treatment Confers Protection against Influenza A Virus Infection

**Fig. 3.**
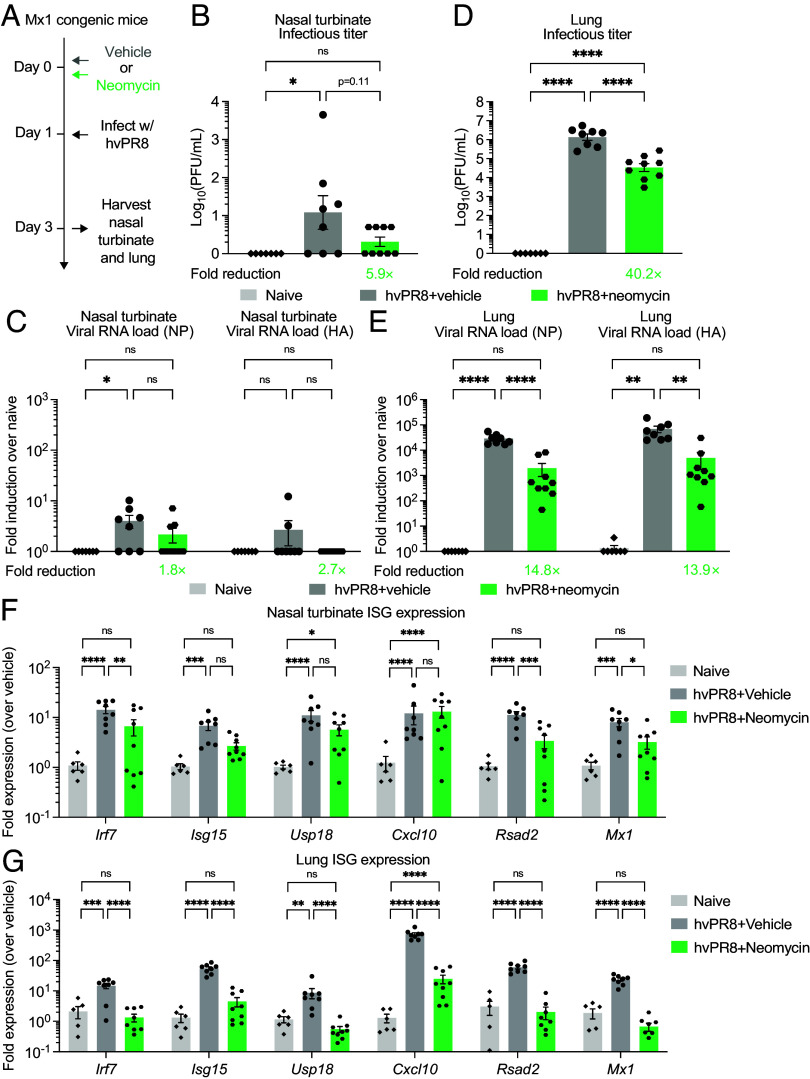
Neomycin confers respiratory protection against influenza A virus infection. (*A*) Experimental schema. Mx1 congenic mice housed in a SPF facility were intranasally treated once with 2 mg neomycin or vehicle delivered in 25 μL volume per nostril. One day after treatment, mice were intranasally infected with 26.5 PFU highly virulent PR8 (hvPR8). On 2 DPI, nasal turbinate and lung tissues were collected for viral titer analysis 2 DPI. (*B*) Measurement of infectious virus titer in the nasal turbinate at 2 DPI by plaque assay (Naïve, n = 7; hvPR8 + Vehicle, n = 8; hvPR8 + Neomycin, n = 9). (*C*) Measurement of vRNA in the nasal turbinate at 2 DPI by RT-qPCR against influenza nucleoprotein (NP) or hemagglutinin (HA) genes (Naïve, n = 7; hvPR8 + Vehicle, n = 8; hvPR8 + Neomycin, n = 9). (*D*) Measurement of infectious virus titer in the lung at 2 DPI by plaque assay (Naïve, n = 7; hvPR8 + Vehicle, n = 8; hvPR8 + Neomycin, n = 9). (*E*) Measurement of vRNA in the lung at 2 DPI by RT-qPCR against NP or HA genes (Naïve, n = 7; hvPR8 + Vehicle, n = 8; hvPR8 + Neomycin, n = 9). (*F* and *G*) Expression of ISGs *Irf7*, *Isg15*, *Usp18*, *Cxcl10*, *Rsad2*, and *Mx1* in nasal turbinate (*F*) and lung homogenate (*G*) tissues at 2 DPI with influenza hvPR8 in Mx1 congenic mice pretreated with 2 mg of neomycin 24 h before infection. Gene expression was normalized against housekeeping genes *Hprt*, and then compared against biological controls (vehicle-treated mice). Fold reduction was calculated as average fold reduction in infectious (*B* and *D*) or genomic RNA (*C* and *E*) virus load between neomycin-treated groups and vehicle controls. Mean ± SEM; statistical significance was calculated by one-way ANOVA followed by Tukey correction (*B*–*E*) or two-way ANOVA followed by Tukey’s correction (*F* and *G*). Individual data points are represented. Data are pooled from two independent experiments.

To assess whether the antiviral property of neomycin can be extended to other respiratory infection settings, we used an Mx1 congenic mouse model of influenza infection. Most inbred mouse strains do not carry functional copies of Mx1 (16), an ISG that potently restricts influenza virus infection (17). These mice are highly susceptible to influenza infection as they lack Mx1-mediated antiviral resistance. Mx1 congenic mice present a unique opportunity to study the ability of intranasal neomycin in influenza protection (18). We infected neomycin-pretreated Mx1 congenic mice with a highly virulent strain of influenza A virus A/PR8 (hvPR8), which causes lethal infection even in the presence of Mx1 (19) (Fig. 3*A*). We have previously shown that intranasal neomycin treatment induces ISG expression in the lung and improves survival in hvPR8-infected Mx1 mice (11). Whether this was due to reduced viral replication in lung tissues was not investigated. Furthermore, it remained unclear whether neomycin exerts antiviral effects in the upper respiratory tract against hvPR8 infection. We found that a single dose of neomycin was able to reduce the level of viral titers in nasal turbinates, although it did not reach statistical significance due to the overall low degree of viral replication (Fig. 3 *B* and *C*). Given that hvPR8 did not replicate well in the upper respiratory tract, we also assessed neomycin-mediated protection against hvPR8 infection in the lower respiratory tract. We accordingly increased the inoculation volume of neomycin from 10 to 25 μL per nostril to ensure neomycin can be adequately delivered to the lower respiratory tract. Notably, neomycin treatment resulted in substantial reduction of RNA viral titers in lung tissues compared to vehicle controls (Fig. 3 *D* and *E*).

In addition to viral loads, we also measured the ISG expressions in the same nasal turbinate and lung tissues. While both infectious titers and genomic viral RNA levels were barely detectable in nasal turbinate tissues infected with the hvPR8 virus, we observed robust induction of ISGs compared to naive controls (Fig. 3*F*). Consistent with reduced viral load, we found significantly attenuated expression of ISGs in nasal turbinate tissues in neomycin-treated mice compared to vehicle controls, including *Irf7*, *Rsad2,* and *Mx1*, likely resulting from lower viral burden at this time point. There was a trend in reduced levels of *Isg15* and *Usp18*, although the difference did not reach statistical significance. The extent of ISG attenuation at day 3 post infection was even more apparent in the lungs, where all ISGs had significantly reduced levels in neomycin-treated mice compared to vehicle controls (Fig. 3*G*). This again was likely due to the ability of neomycin to promote faster viral clearance in lung tissues following hvPR8 infection. Collectively, our results demonstrate that intranasal treatment of neomycin protects against upper and lower respiratory infection of influenza A virus.

## Intranasal Neomycin Therapy Mitigates Disease and Curbs Infection of SARS-CoV-2

**Fig. 4.**
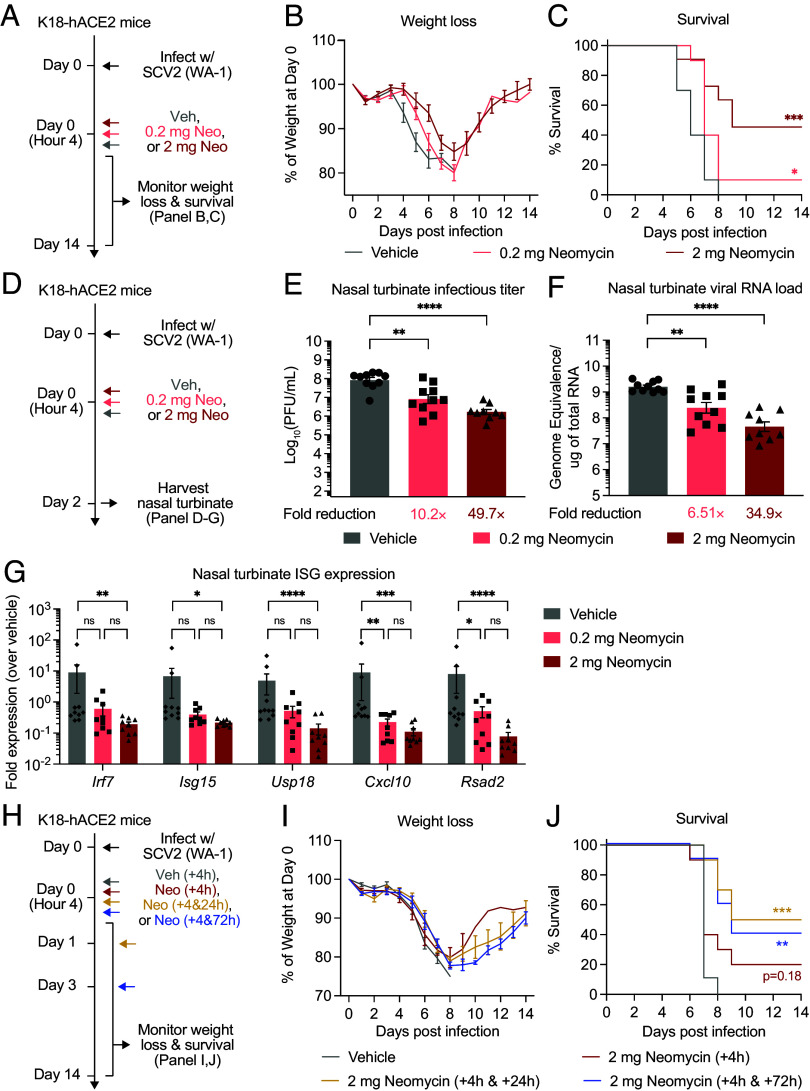
Neomycin mitigates disease progression and curbs infection of SARS-CoV-2. (*A*) Experimental schema. K18-hACE2 mice housed in a SPF facility were intranasally infected with 5 × 10^3^ PFU SARS-CoV-2 (2019n-CoV/USA_WA1/2020). Four hours after infection, mice were intranasally treated once with 0.2 mg neomycin, 2 mg neomycin or vehicle delivered in 10 μL volume per nostril. Weight loss and survival were monitored daily up to 14 DPI. Death was recorded when mice were moribund, or at 80% of original body weight. (*B* and *C*) Weight loss and survival of K18-hACE2 mice therapeutically treated with vehicle, 0.2 mg neomycin, or 2 mg neomycin from 1 to 14 DPI (Vehicle, n = 10; 0.2 mg Neomycin, n = 10; 2 mg Neomycin, n = 11). (*D*) Experimental schema. K18-hACE2 mice housed in a SPF facility were intranasally infected with 5 × 10^3^ PFU SARS-CoV-2 (2019n-CoV/USA_WA1/2020). Four hours after infection, mice were intranasally treated once with 0.2 mg neomycin, 2 mg neomycin or vehicle delivered in 10 μL volume per nostril. Nasal turbinate tissues were collected for viral titer analysis 2 DPI. (*E*) Measurement of infectious virus titer in the nasal turbinate at 2 DPI by plaque assay (Vehicle, n = 10; 0.2 mg Neomycin, n = 10; 2 mg Neomycin, n = 9). Limit of detection (LOD): 10^2^ PFU/mL. (*F*) Measurement of vRNA in the nasal turbinate at 2 DPI by RT-qPCR against SARS-CoV-2 N gene using the CDCN1 primer-probe set (Vehicle, n = 10; 0.2 mg Neomycin, n = 9; 2 mg Neomycin, n = 10). (*G*) Expression of *Irf7*, *Isg15*, *Usp18*, *Cxcl10*, and *Rsad2* in nasal turbinate tissues at 2 DPI from mice infected with 5 × 10^3^ PFU SARS-CoV-2 (2019n-CoV/USA_WA1/2020) and treated with Neomycin at 4 h postinfection. Gene expression was normalized against housekeeping genes *Hprt*, and then compared against biological controls (vehicle-treated mice) (Vehicle, n = 10; 0.2 mg Neomycin, n = 9; 2 mg Neomycin, n = 9), (*H*) Experimental schema. K18-hACE2 mice housed in a SPF facility were intranasally infected with 5 × 10^3^ PFU SARS-CoV-2 (2019n-CoV/USA_WA1/2020). Four HPI, mice were intranasally treated once with 2 mg neomycin or vehicle. Two subsets of neomycin-treated mice were provided with a second dose of 2 mg neomycin on 1 and 3 DPI, respectively. Weight loss and survival were monitored daily up to 14 DPI. Death was recorded when mice were moribund, or at 80% of original body weight. (*I* and *J*) Weight loss and survival of K18-hACE2 mice therapeutically treated with vehicle, neomycin dosed at 4 HPI, 2 mg neomycin dosed at 4 HPI and 1 DPI or neomycin dosed at 4 HPI and 3 DPI from 1 to 14 DPI (Vehicle, n = 9; Neomycin [+4 h], n = 10; Neomycin [+4 h & +24 h], n = 10; Neomycin [+4 h & +72 h], n = 10). Fold reduction was calculated as average fold reduction in infectious (*D*) or genomic RNA (*E*) virus load between neomycin-treated groups and vehicle controls. Mean ± SEM; statistical significance was calculated by the log-rank Mantel–Cox test (*C* and *J*), one-way ANOVA followed by Tukey correction (*E* and *F*) or two-way ANOVA followed by Tukey’s correction (*G*). In weight loss curves, error bars for timepoints with less than 3 alive animals were not shown. Individual data points are represented. Data are pooled from two independent experiments.

Treatment timing relative to viral exposure determines the protective capacity of antiviral therapeutics (20, 21). To determine whether neomycin treatment produces a therapeutic effect when administered after viral exposure, we intranasally treated SARS-CoV-2 infected mice with 2 or 0.2 mg of neomycin 4 hour post infection (HPI) (Fig. 4*A*). Single-dose neomycin therapy delayed weight loss and improved survival following viral infection in a dose-dependent manner, most notably in mice treated with 2 mg of neomycin (Fig. 4 *B* and *C*). Consistent with disease protection, mice therapeutically treated with neomycin had significantly lower infectious virus titers (Fig. 4*D*) and vRNA, suggesting attenuated nasal viral replication (Fig. 4*E*). We additionally measured ISG expressions in these infected nasal turbinate tissues (Fig. 4*A*). The expression of all ISGs assessed in our panel had significantly reduced levels in mice receiving high-dose neomycin therapy compared to vehicle controls (Fig. 4*F*). Similar trends were observed in the low-dose group, although only *Cxcl10* and *Rsad2* reached statistical significance. These results are consistent with the ability of neomycin therapy to improve infection outcome in a dose-dependent fashion. Furthermore, these findings suggest that at least one mechanism by which neomycin therapy confers protection is through amelioration of prolonged ISG activation as a result of high viral burden.

To assess whether neomycin-mediated disease protection could be further enhanced, we provided neomycin-treated mice with an additional dose of neomycin on 1 or 3 DPI (Fig. 4*G*). Compared with single-dose therapy, the two-dose treatment regimen did not provide additional protection against weight loss following SARS-CoV-2 infection (Fig. 4*H*). However, repeated dosing of neomycin on 1 or 3 DPI did lead to significant improvement in survival (Fig. 4*I*). These data suggest that intranasal application of neomycin shortly after exposure reduces SARS-CoV-2 infection and disease, the protective benefit of which could be further therapeutically boosted with repeated dosing as late as 3 DPI.

## Intranasal Neomycin Reduces Transmission in a Hamster Model of COVID-19

**Fig. 5.**
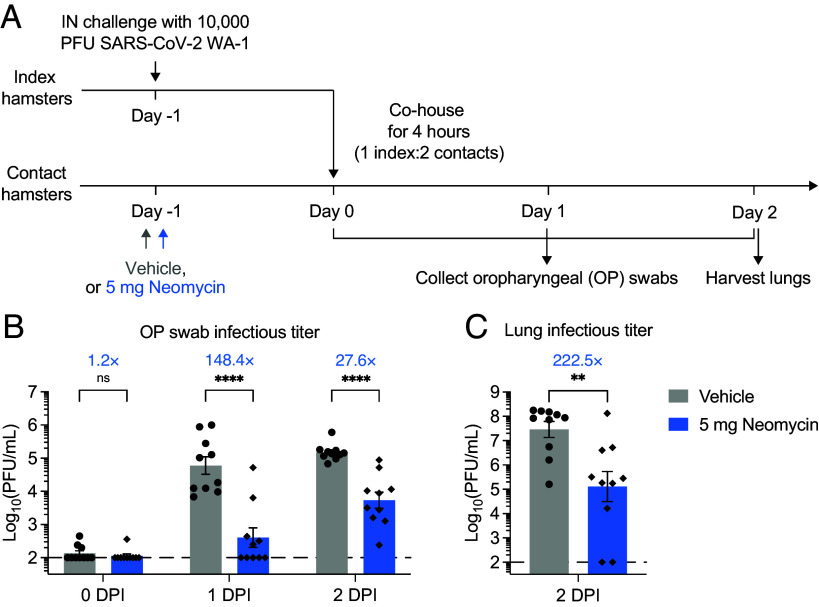
Neomycin reduces contact transmission in a hamster model of SARS-CoV-2. (*A*) Transmission experimental schema. Syrian hamsters were intranasally treated with 5 mg of neomycin or vehicle delivered in 25 μL volume per nostril. Twenty-four hours after neomycin treatment, recipient hamsters were cohoused for 4 h with naïve donor hamsters that had been infected 24 h earlier with 10^4^ PFU SCV2. Daily OP swabs from 0 to 2 DPI as well as lung tissues on 2 DPI were collected. (*B*) Measurement of infectious virus titer in OP swabs on 0 to 2 DPI by plaque assay (Vehicle, n = 10; Neomycin, n = 10). (*C*) Measurement of infectious virus titer in lung tissues on 2 DPI by plaque assay (Vehicle, n = 10; Neomycin, n = 10). Fold reduction was calculated as average fold reduction in infectious (*B* and *C*) virus load between neomycin-treated groups and vehicle controls and indicated above all bar plots. Mean ± SEM; statistical significance was calculated by means of one-way ANOVA followed by Tukey’s correction (*B*) or Student's *t* test (*C*). *P ≤ 0.05, **P ≤ 0.01, ***P ≤ 0.001, ****P ≤ 0.0001. Individual data points are represented. Data are pooled from two independent experiments.

Next, we used Syrian hamsters to assess the ability of neomycin to reduce transmission of SARS-CoV-2. Wild-type Syrian hamsters develop robust respiratory infection upon intranasal SARS-CoV-2 challenge (16, 17). Importantly, SARS-CoV-2 can be transmitted efficiently from infected hamsters to naive hamsters by direct contact and via aerosols. We intranasally treated a cohort of hamsters with 5 mg of neomycin (Fig. 5*A*). Hamsters treated with neomycin were subsequently cohoused with naive donor hamsters that had been infected with SARS-CoV-2 24 h prior. Four hours after cohousing, donor and recipient hamsters were separated, followed by daily collection of oropharyngeal (OP) swabs and terminal lung collection on 2 DPI. Neomycin-treated contact hamsters had significantly lower viral titers in the OP swabs on 1 and 2 DPI after exposure relative to vehicle control animals (Fig. 5*B*). Notably, half of neomycin-treated contact hamsters (5/10) were free of infectious viruses on 1 DPI, although infection was uniformly observed on 2 DPI. The spread of the virus by 2 DPI likely reflects the subsequent transmission of the virus among the cohoused animals. Additionally, neomycin-treated hamsters had significantly reduced viral titers in lung tissues on 2 DPI, with 2 out of 10 hamsters free of infectious viruses (Fig. 5*C*). These results show that intranasal neomycin reduces contact transmission of SARS-CoV-2 in hamsters.

## Intranasal Neosporin Application Induces Variable ISG Induction in Healthy Humans

**Fig. 6.**
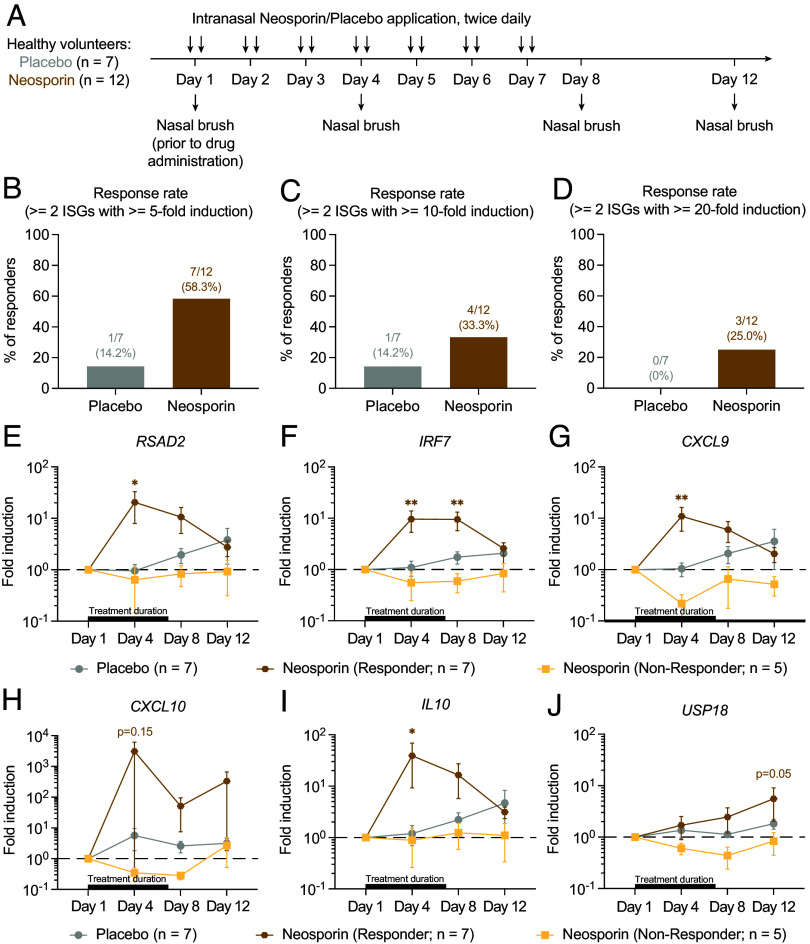
Neosporin application induces ISG expression in the nasal mucosa in healthy humans. (*A*) Experimental schema. Healthy human participants were randomized at a 2:1 ratio into an experimental arm and a placebo arm to receive Neosporin (n = 12) or Vaseline (n = 7) treatment, respectively. Drug application was performed twice-daily for 7 d. Participants had a total of 4 in-person meetings. On days 1, 4, 8, and 12, a nasopharyngeal swab and a nasal brush collection were performed for viral testing and ISG assessment, respectively. (*B*–*D*) Response rate defined as a participant with at least 2 ISGs undergoing at least 5-, 10-, or 20-fold induction at any timepoint compared to day 0 measurements. (*E*–*J*) Expression of *RSAD2*, *IRF7*, *CXCL9*, *CXCL10*, *IL10*, and *USP18* in nasal swab samples. Gene expression was normalized against housekeeping genes *HPRT*, and then compared against day 0 samples. Mean ± SEM; statistical significance was calculated by means of two-way ANOVA followed by Bonferroni’s correction (*E*–*J*); *P ≤ 0.05, **P ≤ 0.01, ***P ≤ 0.001, ****P ≤ 0.0001.

To assess the translational potential of the intranasal neomycin approach in humans, we conducted a small pilot randomized, double-blind, placebo-controlled study involving healthy human participants (Fig. 6*A* and *SI Appendix*, Fig. S2 and *Supporting Text*). For the experimental arm (n = 12), we chose Neosporin ointment, which contains neomycin sulfate, bacitracin, and polymyxin B as its active ingredients. Neosporin is readily available over the counter (OTC) around the world and is currently being used for the clearance of nasal bacterial colonization as well as for management of nosebleeds. For the placebo arm (n = 7), pharmacy-procured Vaseline (Unilever) was used. Participants enrolled in the study were instructed to self-apply Neosporin or Vaseline to the inside of both nostrils using a cotton swab twice-daily for 7 d. On days 1, 4, 8, and 12, nasal brushes were collected from the participants, from which ISG expression was measured by RT-qPCR. A total of six targets were evaluated, including ISGs *RSAD2*, *IRF7*, *CXCL9*, *CXCL10,* and *USP18*, as well as an immunoregulatory cytokine *IL10* known to act downstream of ISG induction (18). During each visit, a nasopharyngeal swab was also collected to monitor signs of infection using a PCR-based clinical respiratory viral panel. None of the samples tested positive for common viral pathogens, including SARS-CoV-2, respiratory syncytial virus, rhinovirus, and influenza A virus, throughout the study. One out of 21 participants experienced signs of an adverse event after 2 doses which self-resolved after a few days and self-withdrew from the study early. Upon examination of their medical record, it was found that the participant had a history of allergic reactions to various medications (not specific to study drug). Another participant’s nasal samples could not be used due to technical issues. All other 19 participants tolerated the treatment well and did not experience any adverse events from the study. Complete demographic features are reported in *SI Appendix*, Table S1.

To assess the overall response rate to Neosporin treatment, we first defined responder (R) as any participant who had at least 2 ISGs being upregulated at least fivefold at any timepoint (day 4, 8, or 12) compared to baseline measurements at day 1. Based on this criterion, we found that Neosporin-treated participants had a much higher response rate compared with placebo controls, 58.3% vs. 14.2%, with 7 out of 12 participants qualifying as R (Fig. 6*B*). This finding held true when the cutoff of ISG fold induction increased from 5 to 10 and 20, with the Neosporin treatment group consistently containing a higher fraction of R (Fig. 6 *C* and *D*). We next analyzed the longitudinal profile of ISG expression, stratifying the Neosporin group based on responder status using the fivefold induction cutoff. Daily Neosporin application resulted in robust upregulation of *RSAD2*, *IRF7*, *CXCL9*, *CXCL10*, and *IL10* in the responder group (Fig. 6 *E*–*I*). The expression peaked on day 3 posttreatment and gradually returned to baseline levels on day 12. In contrast, none of these ISGs were found to be upregulated in nonresponders (NR) from the experimental arm or placebo-treated controls. Neosporin also promoted the expression of *USP18* in the responder group, although the induction was more delayed (Fig. 6*J*). Collectively, these results demonstrate the safety and feasibility of topical neomycin antibiotics as a safe and effective strategy to elicit nasal ISG responses in humans.

## Discussion

This study examined the possibility of using a generic, cheap, and widely available antibiotic neomycin to elicit antiviral activity in the nasal cavity in multiple species. In our previous study, we found a surprising ability of neomycin and other aminoglycoside antibiotics to induce ISGs in vaginal and lower respiratory mucosa in a microbiota-independent manner (11). Building on these findings, in this study we found that intranasally administered neomycin also induced marked ISG upregulation in the upper respiratory tract, similarly independent of the commensal microbiome. We applied these findings to evaluate whether neomycin-triggered ISGs could confer upper respiratory protection against a newly emerged respiratory viral pathogen SARS-CoV-2 in multiple animal models. Importantly, we now provide evidence that elicitation of ISG responses in the upper airway by neomycin protects against SARS-CoV-2 infection in mice and contact transmission in hamsters. Furthermore, we conducted a human study in which we found that intranasal application of Neosporin, an OTC topical antibiotic, induced ISGs in the nose in a subset of healthy humans.

Current antivirals used to treat respiratory viral infections are selective inhibitors that act directly on viral proteins (19). However, targeting viruses directly drives the emergence of drug-resistant strains due to the high natural mutation rate of respiratory viral pathogens. This is exemplified by the emergence of adamantane-resistant A (H3N2), oseltamivir-resistant seasonal A (H1N1) viruses, and adamantane-resistant pandemic A (H1N1) viruses (20). In addition, remdesivir resistance mutations have been observed in COVID-19 patients receiving treatment with the antiviral agent (21). In order to circumvent the emergence of drug-resistant viruses, host-directed therapeutic strategies that engage multiple antiviral mechanisms should be considered. One such strategy is IFN-based therapies, which afford antiviral protection by eliciting ISG expression (22–25). The use of biologics like recombinant IFNs (rIFNs) would greatly reduce the likelihood of the development of drug-resistance viruses. The first success with the use of rIFNs as an antiviral strategy came from randomized controlled trials (RCTs) in the late 1980s for the treatment of chronic hepatitis C virus (HCV) infection (22). These trials demonstrated that polyethylene glycol (PEG)-modified IFNα plus ribavirin could achieve sustained antiviral responses in about half of patients with chronic HCV infection, providing curative treatment for this disease. More recently, multiple IFN-based clinical trials for the treatment of COVID-19 have been conducted, with mixed outcomes depending on stages of treatment administration, IFN types, and delivery formats (26). In a phase 2 RCT, treatment with inhaled nebulized IFN beta-1a led to more rapid recovery in patients admitted to hospital with COVID-19 symptoms compared with placebo treatment (27). In another phase 3 RCT, subcutaneous injection of pegylated IFN lambda among vaccinated outpatients with COVID-19 reduced the risk of hospitalization and emergency department visits compared with placebo treatment (28). Besides rIFN-based approaches, small-molecule agonists that activate innate immune receptors, such as retinoic acid-inducible gene-I-like receptors and toll-like receptors (TLRs), have the potential to be translated into intranasal antiviral therapeutics due to their ability to induce IFN production at mucosal sites (23, 29).

While IFN-based therapy has the potential to be used as an effective treatment option for viral infections, there are several limitations. Systemically injected rIFNs often result in adverse effects that are poorly tolerated, including flu‐like symptoms, hematologic abnormalities, neuropsychiatric disorders, and autoimmune syndromes. This has been well documented in the use of rIFNs for the treatment of chronic HCV infection (30). The manufacturability of biologics such as rIFNs can be complex and challenging. Consequently, direct medical costs range between $1,120 and $1,962 for the IFN treatment regimen and between $2,156 and $5,887 for the PEG-IFN treatment regimen (31, 32). Biologics such as rIFNs also pose significant cold chain requirements. These limitations greatly hindered biologics like rIFNs from being broadly distributed around the world, especially to developing countries with limited infrastructures. In addition, administration of rIFN has been shown to induce neutralizing antidrug antibodies (ADAs) against IFNs that could render the therapy ineffective (33, 34).

In contrast, the intranasal use of Neosporin poses minimal risks as Neosporin has already been extensively available OTC used for clearing nasal bacterial colonization as well as for managing nosebleeds. Unlike rIFNs and other complex biologics, neomycin is compositionally simple, highly manufacturable, cold chain requirement-free and can be readily distributed around the globe. Since neomycin-induced ISG response does not induce robust IFN-I or IFN-III, it does not suffer from ADAs, and can be applied to individuals with preexisting autoantibodies against one or multiple subtypes of IFNs. In addition, neomycin affords broad-spectrum and variant-proof protection against diverse respiratory viruses, presumably due to its ability to induce a broad array of ISGs. Altogether, neomycin presents a promising strategy that is highly manufacturable and readily accessible to all parts of the world in need of therapeutic solutions to combat respiratory viral infections.

From a drug delivery perspective, there are several advantages associated with intranasal delivery over systemic injection. An intranasal administration approach can increase the local concentration of neomycin while minimizing potential side effects associated with systemic exposure. Local administration of neomycin can bypass the physiological barrier between the circulation and the upper respiratory mucosa, allowing for faster induction of ISGs possibly within hours of administration. Intranasally delivered neomycin could potentially produce a more favorable pharmacokinetic profile, as nasal delivery avoids gastrointestinal and hepatic first-pass effects (35). Finally, local self-administration of neomycin will likely increase patient compliance, as the procedure is more convenient and less invasive compared to systemic administration.

In an effort to better understand the mechanism of neomycin-mediated ISG response, we found that intranasal neomycin treatment did not induce appreciable levels of IFN-I or IFN-III in nasal washes. Additionally, neomycin-induced ISGs were unaffected in mice lacking receptors for either IFN-I or IFN-III. These results showed that the ISGs can occur independently of IFN-I or IFN-III receptor signaling following neomycin treatment. Alternatively, IFNAR and IFNLR could play redundant roles in orchestrating ISG responses in the upper airway. This possibility needs to be carefully examined in future studies with mice deficient in lacking both receptors. These observations were consistent with our previous study where we similarly observed robust induction of ISGs in the vaginal mucosa of *Ifnar*^−/−^ mice following intravaginal neomycin application (11). Instead, neomycin directly triggers ISG expression via an innate signaling pathway that involves TLR3, toll/IL-1-receptor-domain-containing adapter-inducing IFN-β signaling adaptor (TRIF), and IFN regulatory factors 3 and 7 (IRF3/7) in type 1 conventional dendritic cells (cDC1s) (11). Whether neomycin engages a similar innate immune pathway to mediate ISG induction in the respiratory mucosa needs to be carefully dissected in future studies. While the signaling requirement for neomycin-triggered ISG responses has been genetically mapped out, the molecular mechanisms by which neomycin activates the TLR3-TRIF-IRF3/7 pathway in cDC1s remain unknown. Aminoglycoside antibiotics are known for their ability to bind mitochondrial ribosomal RNA (rRNA) (36), which resembles bacterial rRNA, and could be recognized by TLR3 to trigger downstream signaling. In support of this hypothesis, our previous work found that kasugamycin, an aminoglycoside antibiotic, synergizes with Poly(I:C) to amplify ISG expression in a TLR3- and TRIF-dependent manner (11). It is possible that neomycin might be directly taken up by cDC1s in the respiratory mucosa and form complexes with mitochondrial rRNA to activate TLR3 in endosomes. Alternatively, neomycin could be taken up by the respiratory epithelium through receptor-mediated endocytosis to cause cell death (37, 38). Subsequently, the dying epithelial cells could be phagocytosed by cDC1s, which enables epithelial cell-derived neomycin-mitochondrial rRNA complexes to access and activate endosomal TLR3 (39). The precise cellular compartment in which neomycin forms complexes with mitochondrial rRNA and the specific RNA species bound by neomycin warrant future investigations.

In order to translate our preclinical findings in mice and hamsters into humans, we conducted a randomized, double-blind, placebo-controlled study involving a small number of healthy human participants given intranasal application of Neosporin. We demonstrate that repeated application of Neosporin twice-daily for 7 d was safe in all but one participant, with no side effect observed up to 30 d posttreatment. Neosporin application rapidly induced several ISGs, with the expression frequently being 5- to 20-fold higher than baseline levels. While neomycin is likely the primary source of ISG-inducing activities in Neosporin, the contributions from other components, namely polymyxin B and bacitracin, cannot be ruled out. Polymyxin B has been shown to promote IFN-γ production in murine natural killer cells (40), and enhance the cytocidal effect of IFN-γ on a human leukemic cell line (41), although the mechanisms for these activities remain unclear. Additionally, polymyxin B enhances the ability of immunostimulatory sequences such as synthetic CpG-containing oligodeoxynucleotides to trigger IFN-α production in plasmacytoid dendritic cells (42). Whether polymyxin B and bacitracin induce ISG expression in the upper airway directly/indirectly should be formally examined in animal models and humans in future studies. The ISG response induced by Neosporin was variable among the treated participants, which could be due to interindividual biological differences or technical issues such as insufficient application or fast clearance of the drug. It is worth noting that after body weight normalization the total amount of neomycin applied in human participants (a pea-size cream contains approximately 0.875 mg neomycin, equivalent to 0.175 mg/kg at 70 kg body weight for a twice-daily, 7-d treatment regimen) is much smaller compared to that of mice (2 mg; 80 mg/kg at 25 g body weight) or hamsters (5 mg; 50 mg/kg at 100 g body weight). Strategies that aim to improve the response rate and magnitude of ISG responses, such as formulating a Neosporin ointment with higher effective neomycin concentration should be pursued in future studies. Other limitations of this study include a very small sample size, the lack of dose–responses, and direct evaluation of antiviral protection in humans. Future studies are needed to determine the ability of topically applied neomycin to prevent infection and reduce transmission between humans.

Overall, our study highlights a previously unexplored treatment modality that evokes protective antiviral immunity in the upper airway using preclinical animal models and randomized controlled human studies. We envision this simple yet effective strategy to be readily deployed in resource-limited, developing countries to combat respiratory viral diseases.

## Materials and Methods

### Animals.

C57BL/6J (B6J) and B6.Cg-Tg(K18-ACE2)2Prlmn/J (K18-hACE2) mice were purchased from The Jackson Laboratory and subsequently bred and housed at Yale University. C57BL/6 mice carrying functional Mx1 alleles were a gift from P. Staeheli (University Medical Center Freiburg). Syrian hamsters (Strain HSdHan:AURA; stock no. 089) were purchased from Envigo. Animals were fed with regular rodent’s chow and sterilized water ad libitum, and maintained on a 12-h light/dark cycle under SPF conditions. For GF experiments, C57BL/6 mice were maintained in flexible plastic gnotobiotic isolators with a 12-h light/dark cycle and provided with a standard, autoclaved mouse chow ad libitum and autoclaved water. GF status was monitored by culture-based and culture-independent methods. All procedures used in this study (sex-matched and age-matched) complied with federal guidelines and the institutional policies of the Yale School of Medicine Institutional Animal Care and Use Committee (IACUC), under protocol #10365.

### Cells and Viruses.

SARS-CoV-2 isolate hCOV-19/USA-WA1/2020 (NR-52281) was obtained from BEI Resources and was amplified in VeroE6 cells overexpressing ACE2 and TMPRSS2 (kindly provided by B. Graham at the NIH Vaccine Research Center), as previously described. Viral titers were determined with standard plaque assay by using Vero E6 cells overexpressing hACE2 and TMPRSS2. SARS-CoV-2 omicron variants were isolated from nasopharyngeal swabs of infected individuals. Samples belonging to the BA2.12.1, BA.4, and BA.5 lineages were selected for virus isolation as previously described. The highly virulent variant of A/PR8 was a gift from P. Staeheli (University Medical Center, Freiburg). Influenza virus strain A/PR/8/34 (H1N1) was propagated as previously described (43). Cell lines obtained from ATCC were not further authenticated and all cell lines were routinely tested for mycoplasma contamination.

### Virus Infection.

Before infection, mice were anesthetized using a mixture of ketamine and xylazine injected intraperitoneally. K18-hACE2 mice were intranasally infected with 5 × 10^3^ PFU SARS-CoV-2 in 50 µL. For flu infection, mice were infected with 26.5 PFU highly virulent A/PR8 influenza strain in 50 µL. Hamsters were anesthetized by using 30% v/v isoflurane diluted in propylene glycol and administered 10^4^ PFU SARS-CoV-2 intranasally in 100 μL. Experiments involving SARS-CoV-2 infection were performed in a biosafety level 3 facility with approval from the Yale Institutional Animal Care and Use Committee and Yale Environmental Health and Safety.

### Antibiotic Treatment.

Neomycin sulfate (Gibco) was dissolved in sterile water at 100 mg/mL stock concentration and stored at −80 °C. Additional dilution was performed in sterile water if needed. Water was used as a vehicle control. Before treatment, mice were anesthetized using 30% (vol/vol) isoflurane diluted in propylene glycol. A total 20 μL of antibiotic was administered dropwise into the nasal cavity using a pipette tip, with each nostril receiving 10 μL of antibiotic. For hamsters, a total 50 μL of antibiotic was administered dropwise into the nasal cavity using a pipette tip, with each nostril receiving 25 μL of antibiotic. For influenza infection experiments using Mx1 mice, antibiotic was administered in 50 μL volume (25 μL/nostril).

### Measurements of SARS-CoV-2 Genomic RNA and Infectious Virus.

Mice were euthanized in 100% isoflurane at indicated time points following the IACUC protocol #10365. Nasal tissues were isolated by removing the head from the mouse body, then discarding the lower mandible from the snout. The skin was subsequently removed to expose the bone of the entire skull and nose. The remaining head was cut in half sagittally, with all tissues caudal to the cribriform plate removed. vRNA and infectious titer from mouse nasal tissues were measured as previously described (44). Nasal tissues were placed in a bead homogenizer tube containing 1 mL of PBS and stored at −80 °C. Nasal turbinate homogenates were cleared of debris by centrifugation (15 min, 3,100 g). Daily oral swabs (Pruitan PurFlock Ultra 25-3206-U) were performed on hamsters and stored in 1 mL of Dulbecco's Modified Eagle Medium (DMEM) with 2% Fetal Bovine Serum (FBS) and 2% antibiotics/antimycotics (Gibco) and stored at −80 °C. To determine infectious SARS-CoV-2 titers, plaque assay was performed by using ACE2- and TMPRSS2-overexpressing Vero E6 cells. Plaques were resolved by means of formalin fixation 40 to 42 h after infection, followed by staining with crystal violet for plaque visualization. For viral RNA analysis, 250 µL of the nasal turbinate homogenates was added to 750 µL Trizol LS (Invitrogen), and RNA was extracted with the RNeasy Mini Kit (Qiagen) according to the manufacturer’s instructions. SARS-CoV-2 RNA levels were quantified with 500 ng RNA inputs using the Luna Universal Probe One-Step RT-qPCR Kit (New England Biolabs) using the real-time RT-PCR primer/probe set 2019-nCoV_N1 (CDCN1).

### Measurements of Influenza Genomic RNA and Infectious Virus.

At indicated time points, mice were euthanized with 100% isoflurane. The whole lung or nasal turbinate was placed in a Lysing Matrix D tube (MP Biomedicals) with 1 mL PBS and homogenized using a table-top homogenizer at medium speed for 2 min. Tissue homogenates were cleared of debris by centrifugation (15 min, 3,100 g). To determine infectious influenza viral titers, plaque assay was performed by standard protocol using Madin-Darby canine kidney (MDCK) cells. Briefly, plaques were resolved by crystal violet staining 48 to 72 h after infection and rinsing with water for plaque visualization. For viral RNA analysis, 250 µL of the nasal turbinate or lung homogenates was added to 750 µL Trizol LS (Invitrogen), and RNA was extracted with the RNeasy Mini Kit (Qiagen) according to the manufacturer’s instructions. The extracted RNA was reverse transcribed into cDNA using the iScript cDNA synthesis kit (Bio-Rad). Influenza A/PR8 RNA levels were quantified with 100 ng cDNA inputs using the iTaq Universal SYBR Green Supermix (Bio-Rad) and the following primers (5′-3′): PR8 hemagglutinin (Forward: AGTGCCCAAAATACGTCAGG, Reverse: GGCAATGGCTCCAAATAGAC), PR8 nucleoprotein (Forward: AGAACATCTGACATGAGGAC, Reverse: GTCAAAGGAAGGCACGATC).

### Measurements of Mouse Gene Expression by RT-qPCR.

Following RNA extraction, total cDNA was prepared with 2 µg RNA inputs using the iScript cDNA Synthesis Kit following the manufacturer’s instruction (Bio-Rad). RT-qPCR was then performed using the SYBR Green PCR Master Mix (Applied Biosystems). For each RT-qPCR, 100 ng cDNA input was used. The following primers were used for mouse ISG expression *Hprt* (Forward: TCAGTCAACGGGGGACATAAA, Reverse: GGGGCTGTACTGCTTAACCAG); *Isg15* (Forward: GCTAGAGCCTGCAGCAATG, Reverse: CCAATCTTCTGGGCAATCTGC); *Irf7* (Forward: TGTAGACGGAGCAATGGCTGAAGT, Reverse: ATCCCTACGACCGAAATGCTTCCA); *Usp18* (Forward: CGTGCTTGAGAGGGTCATTTG, Reverse: GGTCCGGAGTCCACAACTTC); *Cxcl10* (Forward: AGAATGAGGGCCATAGGGAA, Reverse: CGTGGCAATGATCTCAACAC); *Rsad2* (Forward: AACAGGCTGGTTTGGAGAAG, Reverse: TGCCATTGCTCACTATGCTC); *Mx1* (Forward: GACCATAGGGGTCTTGACCAA, Reverse: (AGACTTGCTCTTTCTGAAAAGCC). RT-qPCRs were run in duplicates. The duplicate Ct values were averaged, normalized against housekeeping genes *Hprt* (for mouse) or *GAPDH* (for human), and then compared against biological controls (untreated mice) using the ΔΔCt method of comparison. Fold expression was calculated assuming a doubling efficiency (2) per cycle (fold expression = 2^–ΔΔCt^).

### Immunohistochemistry.

The head was fixed in a 4% paraformaldehyde buffer overnight at 4 °C, then transferred into 1 M ethylenediaminetetraacetic acid (EDTA) solution (Invitrogen) at pH 8.0 for 7 d at 4 °C, changing the EDTA solution once on day 4. After decalcification, Yale Pathology Tissue Services (YPTS) performed formalin fixed paraffin embedding, sectioning, and immunohistochemical staining of nasal tissues. For SARS-CoV-2 N protein staining, polyclonal rabbit-derived IgG (GeneTex; GTX135357) were used at recommended dilutions. Stained sections were then imaged by a fluorescence microscope (BX51; Olympus) with a 10× lens.

### RNA FISH.

RNA ISH was performed using the RNAScope kit (Bio-Techne, Cat # 323100) following the manufacturer’s instructions. Decalcified tissues were embedded in paraffin and sliced into coronal cross-sections using a microtome. Slices were mounted on a glass slide. Slides were first deparaffinized, treated with hydrogen peroxide blocking step, and then with protease prior to using the RNAScope target antigen retrieval. Probe hybridization and amplification steps were followed per manufacturer protocol. Slides were stained with DAPI to visualize nuclei and mounted on slides using ProLong Gold Antifade (ThermoFisher, Cat # P36934). The *Cxcl10* probe was purchased from Bio-Techne (Cat # 408921). Opal 690 Reagent (Akoya Biosciences, Cat # FP1497001KT) was used to visualize staining using a Stellaris 8 confocal microscope (Leica).

### Determination of Determination of IFN-I, IFN-II, IFN-III, and IL-1α Concentration in Nasal Washes.

Mice were euthanized with 100% isoflurane following the IACUC protocol #10365. After euthanasia, the head was removed from the mouse body, and the lower mandible was discarded from the snout. Nasal wash was performed by pushing 500 μL of PBS using a plastic tube attached to a 26-gauge needle through the nasopharynx into a 1.5 mL Eppendorf tube. Nasal washes were repeated 3 times. Samples were centrifuged at 3,900 rpm for 5 min at 4 °C; the supernatant was aliquoted in 100 µL aliquots and stored at −80 °C. Concentration of IFN-I in nasal washes was determined by ELISA (42120 and 42400; PBL Assay Science) as previously described. Concentration of IFN-λ in nasal washes was determined by ELISA (DY1789B; R&D Systems) as previously described. Concentration of IFN-γ in nasal washes was determined by ELISA (KMC4021; Invitrogen) according to the manufacturer’s instructions. Concentration of IL-1α in nasal washes was determined by ELISA (BMS611; Invitrogen) according to the manufacturer’s instructions.

### Graphical Illustrations.

Graphical illustrations were made with Biorender.com.

## Supplementary Material

Appendix 01 (PDF)

Dataset S01 (DOCX)

## Data Availability

All study data are included in the article and/or supporting information.
