# Supplementary material for: Intranasal neomycin evokes broad-spectrum antiviral immunity in the upper respiratory tract
Source: Proc Natl Acad Sci U S A. 2024 Apr 22;121(18):e2319566121. doi: 10.1073/pnas.2319566121 (PMC11067057; doi:10.1073/pnas.2319566121)
Supplement: Supplementary file 1 — Appendix 01 (PDF) [file pnas.2319566121.sapp.pdf]

PNAS

Supporting Information for

Intranasal neomycin evokes broad-spectrum antiviral immunity in the upper respiratory tract

Tianyang Mao[a,1], Jooyoung Kim[b,1], Mario A. Peña-Hernández[a,c,1], Gabrielee Valle[b], Miyu Moriyama[a], Sophia Luyten[a], Isabel M. Ott[a], Maria Luisa Gomez-Calvo[a], Jeff R Gehlhausen[d], Emily Baker[d], Benjamin Israelow[a,e], Martin Slade[f], Lokesh Sharma[b], Wei Liu[b], Changwan Ryu[b], Asawari Korde[b], Chris J. Lee[b], Valter Silva Monteiro[a], Carolina Lucas[a], Huiping Dong[a], Yi Yang[a], Yale SARS-CoV-2 Genomic Surveillance Initiative[2], Smita Gopinath[g], Craig B. Wilen[a,h], Noah Palm[a], Charles S. Dela Cruz[b,c,i*], Akiko Iwasaki[a,d,j,k*]

[a]Department of Immunobiology, Yale University School of Medicine, New Haven, CT, USA.
[b]Department of Internal Medicine, Section of Pulmonary, Critical Care and Sleep Medicine, Yale University School of Medicine, New Haven, CT, USA.
[c]Department of Microbial Pathogenesis, Yale School of Medicine, New Haven, CT, USA.
[d]Department of Dermatology, Yale School of Medicine, New Haven, CT, USA.
[e]Department of Internal Medicine, Section of Infectious Diseases, Yale University School of Medicine, New Haven, CT, USA.
[f]Department of Internal Medicine, Section of Occupational Medicine, Yale University School of Medicine, New Haven, CT, USA.
[g]Department of Immunology and Infectious Diseases, Harvard T.H. Chan School of Public Health, Boston, MA, USA.
[h]Department of Laboratory Medicine, Yale School of Medicine, New Haven, CT, USA.
[i]Veterans Affairs Medical Center, West Haven, CT, USA.
[j]Center for Infection and Immunity, Yale School of Medicine, New Haven, CT, USA.
[k]Howard Hughes Medical Institute, Chevy Chase, MD, USA.

[1]These authors contributed equally to this work.
[2]A full list of authors appears at the end of the paper.
*Correspondence to: Charles S. Dela Cruz and Akiko Iwasaki.
Emails: charles.delacruz@yale.edu and akiko.iwasaki@yale.edu.

This PDF file includes:

Supporting text
Figures S1 to S2
Tables S1
Complete list of the Yale SARS-CoV-2 Genomic Surveillance Initiative authors

Supporting Text

Supplementary Methods

Ethics. This study was approved by the Yale Institutional Review Board (IRB #2000032248 "Topical Antibacterial Agents for Prevention of COVID-19"). All necessary participant consent has been obtained and the appropriate institutional forms have been archived.

Human study design. A randomized control study involving healthy participants was carried out to assess whether intranasal application of neomycin could induce nasal ISG responses in human participants (**Supplementary Fig 2**). For the experimental arm, intranasal antibiotic to be used was Neosporin, which contains neomycin sulfate, bacitracin and polymyxin B at the dose 3.5 mg, 400 units and 5000 units per gram, respectively. For the placebo arm, pharmacy procured Vaseline (Unilever) was used. Neosporin and Vaseline were obtained from pharmacy and handled and distributed by Yale Investigational Pharmacy. The study included 19 participants, 12 receiving Neosporin and 7 receiving placebo. During the course of the study, participants were instructed to apply a small amount of the Neosporin or Vaseline cream (less than a pea size) to the inside of one nostril and repeat for the other nostril using a cotton swab. Participants were then to pinch the nose to spread the cream. Drug application was performed twice daily, once in the morning and once in the evening, for 7 days. Participants had a total of 4 in-person meetings. On day 1, participants had a review of study protocol and signing of consent form. Nasopharyngeal swab and nasal brush were performed for baseline measurement prior to drug administration. Participants were instructed on the application of Neosporin versus placebo. Participants were blinded from the study drug. On days 4, 8, and 12, participants returned to have nasopharyngeal swab and nasal brush performed. During each visit, the study team reviewed for any unexpected adverse events from the study, including but are not limited to nasal congestion, swelling, nasal or skin irritation, or hearing loss. Nasopharyngeal swab and nasal brush samples collected at each timepoint were received by the Yale laboratory for assessment of respiratory viral infections (including SARS-CoV-2, respiratory syncytial virus, rhinovirus and influenza A virus) and nasal ISG response, respectively. On day 30, an additional phone consultation was arranged to review adverse events arising from the study.

Enrollment strategy. Participants were recruited via IRB-approved advertisements delivered through study flyers located in hospital public spaces and on social media platforms, in collaboration with Yale Center for Clinical Investigation. Informed consent was provided by all participants at the time of enrollment. Participants were recruited to Yale Church Street Research Unit in New Haven for study instructions and biospecimen collection by trained research staff. Enrollment started on October 10th, 2022 and the last patient completed their 30 day visit on December 6th, 2022.

Inclusion/exclusion criteria. To be included in the study, participants met the following criteria. Inclusion criteria include: 1) male and female adults above age 18 – 80 years old, (2) completion of written informed consent, (3) COVID-negative within 48 hours of enrollment based on PCR or antigen test. Exclusion criteria include: (1) patient with active nasal or respiratory symptoms, (2) patient with active or chronic respiratory nasal or respiratory infections and or is currently on antibiotics, (3) patient who has been treated with oral or topical antibiotics with the past 14 days, (4) patient with known allergic history to Neosporin (allergic history to neomycin or bacitracin or polymyxcin), (5) patient with known allergies to aminoglycoside antibiotics (neomycin, tobramycin, gentamycin, others), and (6) patient who is known to currently be pregnant.

Biospecimen processing. Biospecimens were received by the Yale laboratory. Nasopharyngeal swab was processed by collecting specimen medium fluid. Nasal brushes (Celletta TM Brush cell collector) were used to collect nasal cells and resuspended in PBS and stored in cell preserving medium at -80°C. RNA was extracted with the RNeasy Mini Kit (Qiagen) according to the manufacturer's instructions.

Detection of human respiratory pathogens in nasopharyngeal samples. For detection of SARS-CoV-2 from human participant nasopharyngeal samples, the cobas® SARS-CoV-2 Test

(Roche) was used according to the manufacturer's instruction. For detection of other common respiratory viruses from human participant nasal samples, an RT-qPCR-based approach was used. The following primers were used for RSV (Forward: AGATCAACTTCTGTCATCCAGCAA, Reverse: TTCTGCACATCATAATTAGGAGTATCAAT); rhinovirus (Forward: CAGGCCAAATTAAAGTCAATAAGC, Reverse: AGGCTGAAGTTTGGTTTTGC); influenza A virus (Forward: ACAAGACCAATCCTGTCACCT, Reverse: TGGACAAAGCGTCTACGCT).

Measurements of human gene expression by RT-qPCR. Following RNA extraction, total cDNA was prepared with 2 µg RNA inputs using the iScript cDNA Synthesis Kit following the manufacturer's instruction (Bio-Rad). RT-qPCR was then performed using the SYBR Green PCR Master Mix (Applied Biosystems). For each RT-qPCR reaction, 100 ng cDNA input was used. The following primers were used for human ISG expression: *GAPDH* (Forward: GTCTCCTCTGACTTCAACAGCG, Reverse: ACCACCCTGTTGCTGTAGCCAA); *IRF7* (Forward: GCTGGACGTGACCATCATGTAC, Reverse: GGGCCGTATAGGAACGTGC); *USP18* (Forward:GGCTCCTGAGGCAAATCTGT, Reverse: CAACCAGGCCATGAGGGTAG); *CXCL10* (Forward: CCACGTGTTGAGATCATTGCT Reverse: TGCATCGATTTTGCTCCCCT); *RSAD2* (Forward: TTGGACATTCTCGCTATCTCCT, Reverse: AGTGCTTTGATCTGTTCCGTC); *MXA* (Forward: GTTTCCGAAGTGGACATCGCA, Reverse: CTGCACAGGTTGTTCTCAGC); *CXCL9* (Forward: AGTGCAAGGAACCCCAGTAG, Reverse: AGGGCTTGGGGCAAATTGTT). RT-qPCR reactions were run in duplicates. The duplicate Ct values were averaged, normalized against housekeeping genes *Hprt* (for mouse) or *GAPDH* (for human), and then compared against biological controls (untreated mice) using the ΔΔCt method of comparison. Fold expression was calculated assuming a doubling efficiency (2) per cycle (fold expression = $2^{-\Delta\Delta Ct}$).

Statistical analysis. Specific statistical methodology can be found in relevant figure legends and manuscript text. Sample size for the human study was pre-determined through formal power analysis. In order to determine the shape of the ISG response level over time curve and have 90% power to demonstrate a 10% difference in ISG response levels between the intervention and control groups with 95% confidence, assuming a curvilinear relationship between ISG response level and time, a variance of 0.38 in the ISG response level, and a correlation among repeated observations of 0.3, thirty-four (34) subjects will need to complete the study. Allowing for a 10% loss to follow-up, 40 subjects will need to be enrolled in the study. For mouse experiments, comparison of survival outcome between experimental groups was performed using log-rank Mantel–Cox test. Comparison of viral titers between two groups was performed using Student's t-test. Comparison of ISG expression levels, viral titers or IFN levels between more than two groups was performed using one-way ANOVA followed by Tukey's correction for multiple comparison. Analysis of ISG expression kinetics across days 1, 3, 5, 7 was performed for each ISG using two-way ANOVA followed by Tukey's correction for multiple comparison. For the human study, the demographics of the two groups (active and placebo) were compared using Student's t-test for the continuous variable of age and Fisher's Exact test for the categorical variables of sex, age, race and ethnicity. Comparison between responders, non-responders and placebo controls at each timepoint (days 1, 4, 8, and 12) was performed using two-way ANOVA followed by Bonferroni's correction. All statistical tests were performed using the GraphPad software. Statistical significance was defined at the cutoff of 95% level of significance ($\alpha = 0.05$).

Supplementary Figure 1

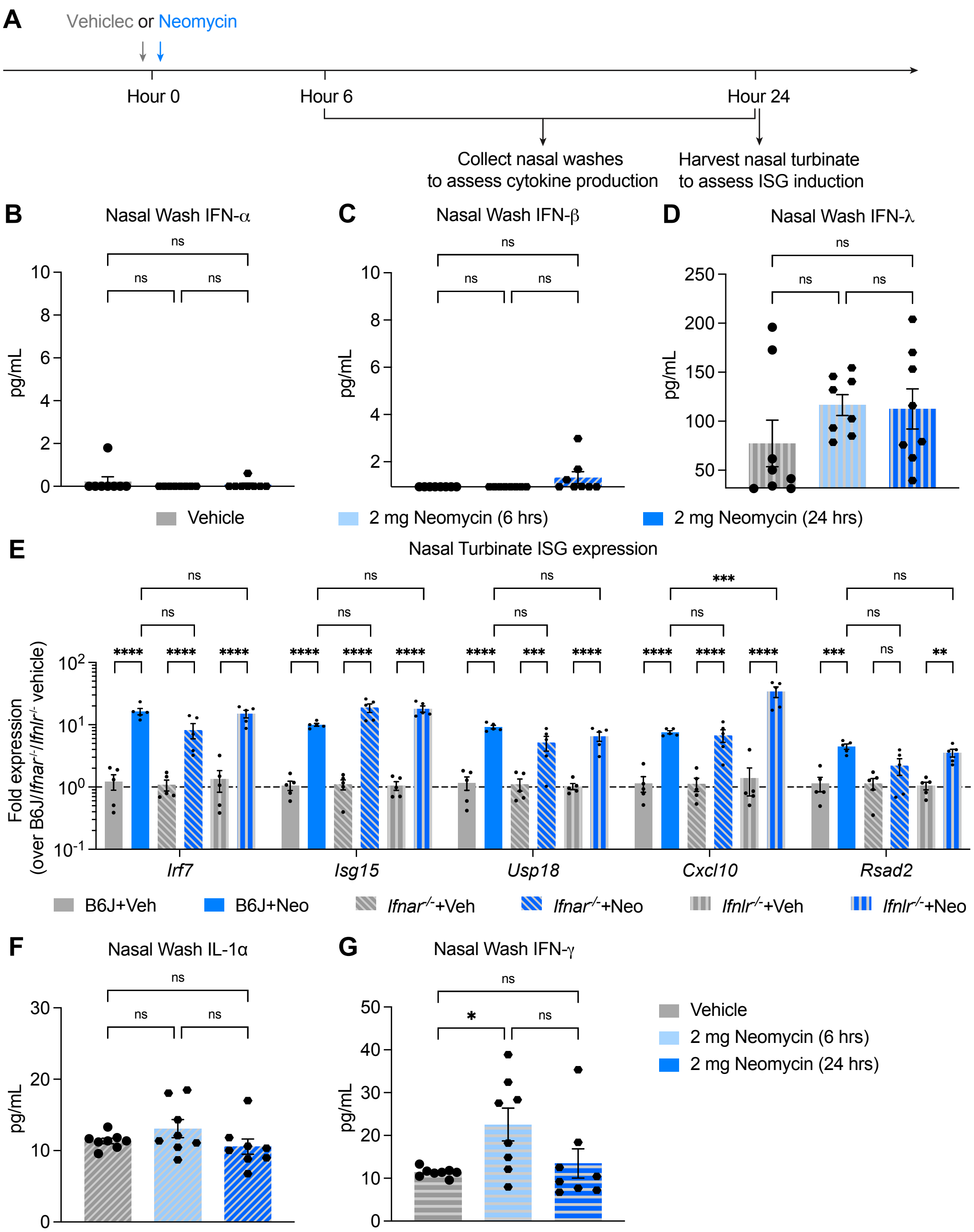

Extended Fig 1: Neomycin-triggered ISG response does not require type I or III IFNs. (**A**) Experimental schema. Specific-pathogen free (SPF) C57BL/6J mice were intranasally treated once with 2 mg neomycin or vehicle. 6 and 24 hrs post treatment, nasal washes were collected for ELISA analysis of IFN-I and IFN-III. 24 hrs post treatment, nasal turbinates were collected for gene expression analysis by RT-qPCR. (**B-D**) Nasal washes from vehicle or neomycin-treated mice were collected for IFN-α, IFN-β and IFN-λ ELISA (Vehicle, n = 8; Neomycin [6 hrs], n = 8; Neomycin [24 hrs], n = 8). (**E**) Expression of ISGs *Irf7*, *Isg15*, *Usp18*, *Cxcl10* and *Rsad2* in nasal turbinate tissues

(B6J + Vehicle, n = 5; B6J + Neomycin, n = 5; *Ifnar*$^{-/-}$ + Vehicle, n = 5; *Ifnar*$^{-/-}$ + Neomycin, n = 5; *Ifnlr*$^{-/-}$ + Vehicle, n = 5; *Ifnlr*$^{-/-}$ + Neomycin, n = 5). Gene expression of B6J, *Ifnar*$^{-/-}$ and *Ifnlr*$^{-/-}$ samples were separately compared to their vehicle controls. (**F,G**) Nasal washes from vehicle or neomycin-treated mice were collected for IL-1α and IFN-γ ELISA (Vehicle, n = 8; Neomycin [6 hrs], n = 8; Neomycin [24 hrs], n = 8). Mean ± SEM; statistical significance was calculated by means of two-way ANOVA followed by Tukey's correction (**B-D, F,G**) or two-way ANOVA followed by Tukey's correction (**E**); *P ≤ 0.05, **P ≤ 0.01, ***P ≤ 0.001, ****P ≤ 0.0001. Individual data points are represented. Data are pooled from two independent experiments.

Supplementary Figure 2

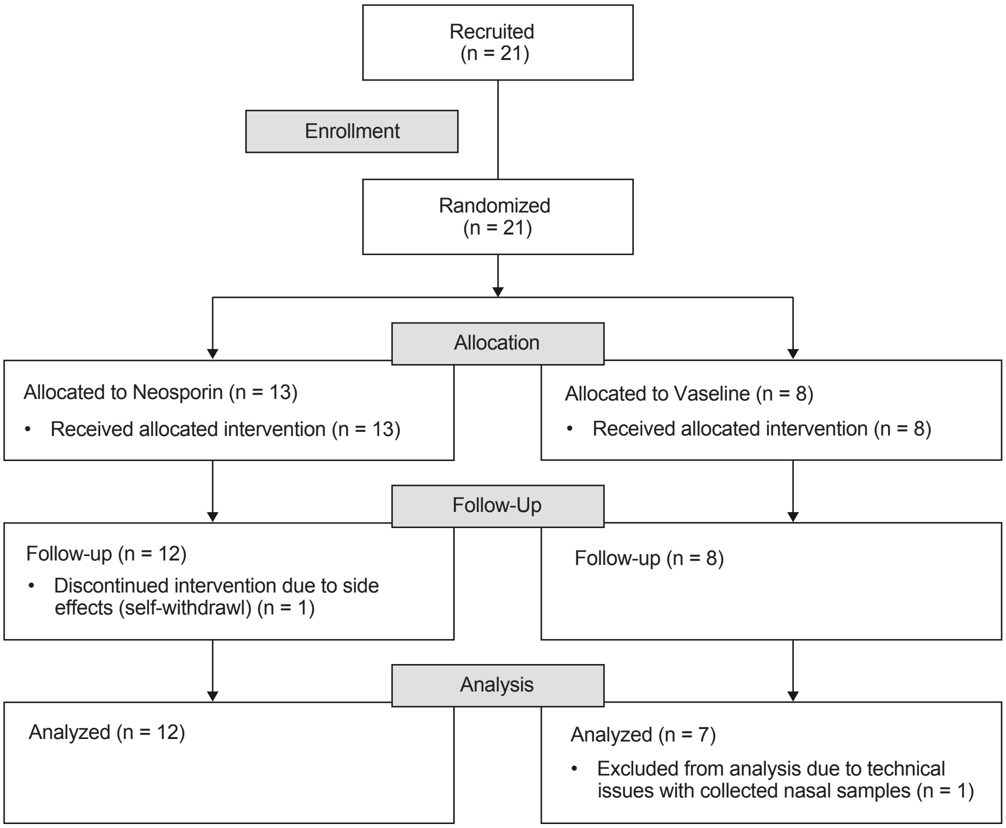

Extended Fig 2: CONSORT flow diagram of the human Neosporin study. To assess whether intranasal application of Neosporin elicits ISG expression in humans, a small pilot randomized, double-blind, placebo-controlled study was conducted. A total of 21 participants were recruited and randomized into an experimental arm receiving Neosporin (n = 13) and a placebo arm receiving Vaseline (n = 8). Participants enrolled in the study were instructed to self-apply Neosporin or Vaseline to the inside of both nostrils using a cotton swab twice daily for 7 days. On days 1, 4, 8, and 12, nasal brushes were collected from the participants, from which ISG expression was measured by RT-qPCR. For the experimental arm, 1 out of 13 participants experienced signs of an adverse event and self-withdrew from the study early. Samples from all remaining 12 participants were analyzed and presented. For the placebo arm, all 8 participants completed the treatment. One out of 8 participants were excluded from the analysis due to technical issues with their nasal samples. Samples from all remaining 7 participants were analyzed and presented.

Table S1: Demographics of the Neosporin human study.

**Demographics**	All	Neosporin	Placebo	Fischer's Exact Test (Two-sided p-value)
Enrolled Participants (n)	19	12	7	
Sex (M | F)	16 3 (84.21% | 15.79%) (n = 19)	11 1 (91.67% | 8.33%) (n = 12)	5 2 (71.42% | 28.57%) (n = 7)	0.5232
Age (years)	38.32 ± 11.31 (n = 19)	36.92 ± 10.18 (n = 12)	40.71 ± 13.54 (n = 7)	0.4961
Race				
Asian	2 (10.53%)	2 (16.67%)	0 (0%)	0.5835
African American	1 (5.25%)	1 (8.33%)	0 (0%)	
White	12 (63.16%)	6 (50.00%)	6 (85.71%)	
Unknown	4 (21.05%)	3 (25.00%)	1 (14.29%)	
Ethnicity				
Hispanic	5 (26.32%)	3 (25.00%)	2 (28.57%)	1
Non-Hispanic	14 (73.68%)	9 (75.00%)	5 (71.43%)	

Summary demographics for the Neosporin human study. Participants were stratified into two arms at enrollment receiving either Neosporin or Vaseline. Various demographic features were reported by row for each arm (row measurement units are specified in parentheses). Within each cell, counts or feature averages are reported, with sample standard deviations, relative cohort percentages, and participant numbers reported where pertinent. Results from statistical tests are reported as Fisher's Exact Test p-value. Abbreviations: n = number; M = male; F = female.

The Yale SARS-CoV-2 Genomic Surveillance Initiative
Chantal B. F. Vogels[1], Anne M. Hahn[1], Nicholas F. G. Chen[1], Mallery Breban[1], Tobias R Koch[1], Chrispin Chaguza[1], Irina Tikhonova[1], Christopher Castaldi[2], Shrikant Mane[2], Bony De Kumar[2], David Ferguson[2], Nicholas Kerantzas[3], David Peaper[3], Marie L Landry[3], Wade Schulz[4], Nathan Grubaugh[5,6]

[1]Yale Institute for Global Health, Yale University, New Haven, CT, USA.
[2]Yale Center for Genome Analysis, Yale University, New Haven, CT, 06510, USA.
[3]Department of Laboratory Medicine, Yale New Haven Hospital, CT 06510, USA.
[4]Center for Outcomes Research and Evaluation, Yale New Haven Hospital, CT 06510, USA.
[5]Department of Epidemiology of Microbial Diseases, Yale School of Public Health, New Haven, CT, USA.
[6]Department of Ecology and Evolutionary Biology, Yale University, New Haven, CT, USA.
