# Supplementary material for: Intranasal neomycin evokes broad-spectrum antiviral immunity in the upper respiratory tract
Source: Proc Natl Acad Sci U S A. 2024 Apr 22;121(18):e2319566121. doi: 10.1073/pnas.2319566121 (PMC11067057; doi:10.1073/pnas.2319566121)
Supplement: Supplementary file 2 — Dataset S01 (DOCX) [file pnas.2319566121.sd01.docx]

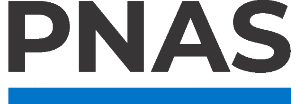


**[SARS-CoV-2 Genomic Surveillance Initiative] Authors**

The following authors were part of the SARS-CoV-2 Genomic Surveillance Initiative:

Chantal B. F. Vogels^1^, Anne M. Hahn^1^, Nicholas F. G. Chen^1^, Mallery Breban^1^, Tobias R Koch^1^, Chrispin Chaguza^1^, Irina Tikhonova^1^, Christopher Castaldi^2^, Shrikant Mane^2^, Bony De Kumar^2^, David Ferguson^2^, Nicholas Kerantzas^3^, David Peaper^3^, Marie L Landry^3^, Wade Schulz^4^, Nathan Grubaugh^5,6^

^1^Yale Institute for Global Health, Yale University, New Haven, CT, USA.

^2^Yale Center for Genome Analysis, Yale University, New Haven, CT, 06510, USA.

^3^Department of Laboratory Medicine, Yale New Haven Hospital, CT 06510, USA.

^4^Center for Outcomes Research and Evaluation, Yale New Haven Hospital, CT 06510, USA.

^5^Department of Epidemiology of Microbial Diseases, Yale School of Public Health, New Haven, CT, USA.

^6^Department of Ecology and Evolutionary Biology, Yale University, New Haven, CT, USA.

Authors from the Yale SARS-CoV-2 Genomic Surveillance Initiative contributed to sample screening, sample processing, viral genome sequencing, and data analysis.
